# Synthesis, Characterization and Cytotoxicity of Novel Thermoresponsive Star Copolymers of *N*,*N*′-Dimethylaminoethyl Methacrylate and Hydroxyl-Bearing Oligo(Ethylene Glycol) Methacrylate

**DOI:** 10.3390/polym10111255

**Published:** 2018-11-12

**Authors:** Barbara Mendrek, Agnieszka Fus, Katarzyna Klarzyńska, Aleksander L. Sieroń, Mario Smet, Agnieszka Kowalczuk, Andrzej Dworak

**Affiliations:** 1 Centre of Polymer and Carbon Materials, Polish Academy of Sciences, M. Curie-Sklodowskiej 34, 41-819 Zabrze, Poland; akowalczuk@cmpw-pan.edu.pl (A.K.); adworak@cmpw-pan.edu.pl (A.D.); 2 Department of Molecular Biology and Genetics, Medical University of Silesia, Medykow 18, 40-752 Katowice, Poland; afus@sum.edu.pl (A.F.); katarzyna.klarzynska@gmail.com (K.K.); alsieron@sum.edu.pl (A.L.S.); 3 Department of Chemistry, University of Leuven, Celestijnenlaan, 200F, B-3001 Leuven (Heverlee), Belgium; mario.smet@kuleuven.be

**Keywords:** star polymers, solution behavior, ATRP

## Abstract

Novel, nontoxic star copolymers of *N*,*N*-dimethylaminoethyl methacrylate (DMAEMA) and hydroxyl-bearing oligo(ethylene glycol) methacrylate (OEGMA-OH) were synthesized via atom transfer radical polymerization (ATRP) using hyperbranched poly(arylene oxindole) as the macroinitiator. Stars with molar masses from 100,000 g/mol to 257,000 g/mol and with various amounts of OEGMA-OH in the arms were prepared. As these polymers can find applications, e.g., as carriers of nucleic acids, drugs or antibacterial or antifouling agents, in this work, much attention has been devoted to exploring their solution behavior and their stimuli-responsive properties. The behavior of the stars was studied in aqueous solutions under various pH and temperature conditions, as well as in PBS buffer, in Dulbecco’s modified Eagle’s medium (DMEM) and in organic solvents for comparison. The results indicated that increasing the content of hydrophilic OEGMA-OH units in the arms up to 10 mol% increased the cloud point temperature. For the stars with an OEGMA-OH content of 10 mol%, the thermo- and pH-responsivity was switched off. Since cytotoxicity experiments have shown that the obtained stars are less toxic than homopolymer DMAEMA stars, the presented studies confirmed that the prepared polymers are great candidates for the design of various nanosystems for biomedical applications.

## 1. Introduction

Although star polymers are often studied as structures for the transport of various bioactive substances [1,2], not enough attention has been devoted to their characteristics in the solutions used in biological experiments. Star macromolecules should ensure good solubility of active substances in aqueous media and protect the substances from undesirable inactivation and decomposition. These requirements are generally satisfied by star structures with a hydrophobic core and a hydrophilic shell. The function of the core is to accept a hydrophobic substance, while the shell ensures the solubility of the nanoparticles in body fluids and protects the transported substance from interactions with other active compounds. The protection of an active biocompound by the star polymer may also be achieved by its complexation with appropriate functional groups of the shell. The coating must also ensure the nontoxicity of the entire system and prevent aggregation with the components of body fluids. Furthermore, the star polymers should be stable in aqueous solutions, which is often enabled by the covalent binding of the core to the arms of the star. To ensure adequate biodistribution, the size of the star polymers is also important. The sizes of such nanoparticles described in the literature for biomedical applications are mostly in the range of 5–200 nm [3,4].

Unfortunately, it is not usually determined if the stars are self-organizing in the presence of salts or biological compounds present in the media or whether a stimulus present in the same temperature or pH range as found in pure water causes the polymers to respond. Moreover, any post-polymerization modification, such as the incorporation of various types of compounds into the structure of stars, e.g., fluorescent [5,6] or targeting moieties [7,8,9], may strongly influence the final solution properties. This fact is often not taken into account in studies. For the above reasons, in our work, we synthesized novel stars with reactive hydroxyl groups that can be used in future applications in biomedicine, and we thoroughly evaluated their properties in aqueous solutions and different media used for biological tests. Herein, we provide the knowledge essential for their intended applications, e.g., for the transport of nucleic acids in gene therapy or for the introduction of appropriate fluorophores to visualize the pathways of stars in the cells via fluorescence microscopy.

Star polymers with arms consisting of *N*,*N*-dimethylaminoethyl methacrylate and oligo(ethylene glycol) methacrylate (OEGMA) with different numbers of ethylene glycol units could be synthesized using different polymerization techniques, e.g., ATRP [9,10,11,12], reversible addition-fragmentation chain transfer (RAFT) [13,14] or group transfer polymerization (GTP) [15]. Both the “core first” and “arm first” approaches have been used to synthesize these structures by the aforementioned polymerizations. In the “core first” method, the multifunctional compound initiates the polymerization of the monomers [9,11,12]. This strategy facilitates the formation of well-defined polymers with precisely controlled numbers of arms and chain lengths. In the “arm first” method, the linear chains have first been synthesized and then crosslinked using a bifunctional coupling agent [10,14,15]. In this way, stars with a very large number of arms have been obtained; however, during core formation, gelation processes may occur and unreacted linear polymer chains may remain in the reaction mixture.

Of the synthesized DMAEMA-*co*-OEGMA star polymers, mainly OEGMA derivatives with methyl or ethyl end groups have been prepared [10,11,12,14,15]. Such stars have been used as nucleic acid carriers in gene therapy, and the cationic amino groups of these polymers condense DNA or RNA into so-called polyplexes, while poly[oligo(ethylene glycol) methacrylate] (POEGMA) segments are responsible for the increase in the biocompatibility [10,12,15]. There are also reports of the use of P(DMAEMA-*co*-OEGMA) stars as drug delivery systems for the transport of doxorubicin [16,17,18] and fluorescein [19]. In most cases, the solution behavior of such stars has not been discussed or the authors focus on the properties of the resulting star complexes with nucleic acids or drugs. In our previous papers, we emphasized the importance of investigating the solution behavior of the prepared stars for the design of effective nanocarriers [11,20,21,22].

The post-polymerization modifications of the abovementioned P(DMAEMA-*co*-OEGMA) stars are limited due to the sterically hindered access to the end groups of the star arms. The use of hydroxyl-bearing OEGMA may be a solution to this problem. To the best of our knowledge, OEGMA-OH has only been used for building stars in the work of Wang [9] who described the synthesis of an eight-armed star copolymer of DMAEMA and OEGMA-OH (*M*_n_ = 360 g/mol) onto a silsesquioxane core using ATRP. The hydroxyl groups of the OEGMA were used to facilitate the further functionalization of the stars with targeting peptides. This report clearly indicated that facile star functionalization is possible with this type of monomer. The authors claimed that those stars self-assemble into micelles with sizes ranging from 128 nm to 242 nm in aqueous solutions, but further studies of the behavior of the stars in solution were not carried out.

The studies performed in this paper are designed to show how the chemical structures of the stars and the ability to control their behavior in various solutions (DMEM, PBS, and organic solvents) as well as in water under various pH and temperature conditions, can guide their future use in biomedical applications. Using the “core-first” method, we synthesized new functional stars with 28 arms made of poly(*N*,*N*-dimethylaminoethyl methacrylate) and hydroxyl-bearing poly[oligo(ethylene glycol) methacrylate]. Hyperbranched poly(arylene oxindole) was used as the star core because of its large number of initiating bromoester groups, leading to a multiarmed star topology. The approach proposed here allowed the relatively simple introduction of reactive groups to the star structure by using functional monomers polymerizable via ATRP. Due to the thermo and pH responsivity of PDMAEMA, for our P(DMAEMA-*co*-OEGMA-OH) stars, we decided to determine how the addition of the OEGMA-OH comonomer would affect their behavior in solution in response to selected stimuli. Thus, the obtained polymers were thoroughly characterized in different solutions under various temperature and pH conditions. For all biomedical applications, the cytotoxicity of the synthesized polymers should be investigated. Hence, the viability of the HT-1080 model cell line in the presence of stars was evaluated. The stars were found to be nontoxic in the concentration ranges required for biological experiments, which allows us to use them in the near future for the preparation of star polymer/nucleic acid complexes and incorporate labeling moieties into their structure for molecular imaging applications.

## 2. Materials and Methods

### 2.1. Materials

*N*,*N*-Dimethylaminoethyl methacrylate (DMAEMA, 99%) was purchased from Merck and purified by distillation prior to use. Hydroxyl-bearing oligo(ethylene glycol) methacrylate with an average molar mass of *M*_n_ = 360 g/mol (OEGMA-OH) was purchased from Sigma Aldrich and purified according to the procedure described in [23]. Branched polyethylenimine (PEI) with *M*_n_ = 25,000 g/mol, 1,2-dichlorobenzene (99%), 1,1,4,7,10,10-hexamethyltriethylenetetramine (HMTETA, 97%), copper (I) bromide (CuBr, 99.999%), copper (II) bromide (CuBr_2_, 99%), p-xylene (99%) and phosphate-buffered saline (PBS) were purchased from Sigma Aldrich and used as received. Dulbecco’s modified Eagle’s medium (DMEM) was also purchased from Sigma Aldrich and was dissolved in sterile distilled water in accordance with the manufacturer’s instructions. DOWEX MARATHON MSC ion exchange resin was purchased from Sigma Aldrich and protonated using 1.6 M HNO_3_. Methanol (99.8%) and tetrahydrofuran (THF, 99.8%) were purchased from POCh and used as received.

Alamar blue reagent (DAL1100) was obtained from Invitrogen and used as a 10% solution. Penicillin/streptomycin/amphotericin B mix, fetal bovine serum (FBS), L-glutamine and trypsin/EDTA (10×) were purchased from PAN Biotech. All reagents were added to the culture medium to proper final concentrations prior to use. L-glutamine was added to the culture medium every two weeks.

### 2.2. Synthesis of Star Polymers with Poly[N,N-Dimethylaminoethyl Methacrylate-co-Hydroxyl-Bearing Oligo(Ethylene Glycol) Methacrylate] Arms (P(DMAEMA-co-OEGMA-OH))

Example star polymer synthesis was as follows: hyperbranched poly(arylene oxindole) (PArOx, 80 × 10^−3^ g, 3.8 × 10^−6^ mol) (synthesis and characterization published elsewhere [24]), was dissolved with CuBr (1.53× 10^−2^ g, 1.07 × 10^−4^ mol) and CuBr_2_ (1.2 × 10^−3^ g, 5.35 × 10^−6^ mol) in 8.6 mL of 1,2-dichlorobenzene in a Schlenk flask under nitrogen with a magnetic stirrer and degassed using freeze-pump-thaw cycles. Then, HMTETA (2.46 × 10^−5^ g, 1.07 × 10^−4^ mol, and 2.9 × 10^−5^ mL) was added to the solution, and the mixture was degassed. The ratio of [PArOx]:[CuBr]:[CuBr_2_]:[HMTETA] was 1:1:0.05:1 in all cases. Next, DMAEMA (1.7 g, 1.08 × 10^−2^ mol, 1.8 mL) in a 1:4 *v*/*v* solution with 1,2-dichlorobenzene was added, and the reaction mixture was degassed twice. The flask was placed in an oil bath at 40 °C. Next, after the desired conversion of DMAEMA, OEGMA-OH (0.39 g, 1.07 × 10^−3^ mol, 0.35 mL, degassed separately) was added to the reaction mixture, which was again thermostated at 40 °C in an oil bath. The monomer-to-initiator ratio was different for each synthesis to obtain stars with arms of different lengths and different OEGMA-OH contents. For details, see Table 1.

The monomers conversion was followed by high-performance liquid chromatography (HPLC) with p-xylene as an internal standard. Samples were taken during the polymerization and analyzed without purification. After the desired molar mass was obtained, THF (5 mL) was added, and the solution was passed through a DOWEX-MSC-1 ion exchange resin column to remove the copper. The resulting solution without copper was dialyzed against methanol and then against water (SpectraPor membrane with MWCO 1000 g/mol) and freeze dried to prevent polymer aggregation.

^1^H NMR (600 MHz, CDCl_3_) *δ_ppm_*: 0.8–1.4 (C*H*_3_C–), 1.7–2.2 (–C*H*_2_C–) in the methacrylate backbone, 2.6–2.8 (–OCH_2_C*H*_2_N–), 4.0–4.2 (–OC*H*_2_CH_2_N–) and (–OC*H*_2_CH_2_O–), 2.2–2.5 (–N-C*H*_3_), 3.6–3.8 (–OC*H*_2_C*H*_2_O–).

### 2.3. Cell Culture

Human HT-1080 fibrosarcoma cells (ATCC#CCL-121) were purchased from American Type Culture Collection (ATCC). Cells were cultured in cell culture medium (CCM) consisting of DMEM supplemented with 10% FBS, 1% L-glutamine, penicillin, streptomycin and amphotericin B, and the cells were cultured at 37 °C under 5% CO_2_.

### 2.4. Cytotoxicity of Star Polymers

One day before the cytotoxicity assays, HT-1080 cells were seeded in 0.5 mL of CCM in 24-well plates at a density of 8 × 10^4^ cells per well. The following day, the CCM was supplemented with tested star polymers and PEI to the desired final concentrations: 0 (control), 5, 10, 20, 30, 40, 50, 60, 70, 80, 90 and 100 µg/mL. The cells were incubated for an additional 24 h, then the medium was removed, and the plates were rinsed with prewarmed PBS buffer. The cell viability was evaluated with the Alamar blue assay. After adding 200 μL of prewarmed Alamar blue reagent in CCM to a final concentration of 10% in each well, the cells were incubated under standard conditions at 37 °C and 5% CO_2_ for 1 h. Subsequently, 100 µL of the mixture from each well was transferred to a new well of a 96-well TPPTM plate (PerkinElmer, Waltham, MA, USA), and the fluorescence emission was monitored at 590 nm using a VICTOR^TM^ Multilabel Plate Reader (PerkinElmer, USA) with a 560 nm excitation source. The cell viability was assessed based on the percent of live cells compared to the control cells not treated with the polymer.

### 2.5. Methods

High-performance liquid chromatography (HPLC) was used to determine the monomer conversion. An Agilent system (1260 Infinity) equipped with an Eclipse XDB-C18 column (84.6 × 150 mm, Agilent) and a UV/VIS diode array detector (Agilent, 1260 DAD VL, Santa Clara, CA, USA) was used. A linear gradient from 10% to 95% B within 40 min was applied (A: 0.1% TFA in water (Gradient Grade, POCh, Poland) and B: 0.1% TFA in acetonitrile (Gradient Grade, 99.9%, POCh, Gliwice, Poland)). The flow rate was maintained at 0.5 mL/min. The chromatograms were recorded at 220 nm using Agilent ChemStation software.

Gel permeation chromatography with multiangle laser light scattering (GPC-MALLS) detection was used to determine the molar masses and molar mass dispersities of the star polymers. The system contained a differential refractive index detector (Δn-2010 RI WGE Dr. Bures) and a multiangle laser light scattering detector (DAWN EOS from Wyatt Technologies, Santa Barbara, CA, USA) and the following columns: GRAM gel guard, GRAM 100 Å, GRAM 1000 Å and GRAM 3000 Å (Polymer Standard Service, Mainz, Germany). GPC was performed in DMF at 45 °C with a nominal flow rate of 1 mL/min. The results were evaluated using ASTRA 5 software from Wyatt Technologies. The refractive index increments (*dn*/*dc*) of the stars were independently measured in DMF using a SEC-3010 *dn*/*dc* WGE Dr. Bures differential refractive index detector.

Dynamic light scattering (DLS) measurements were performed on a Brookhaven BI-200 goniometer (Brookhaven Instruments Corporation, New York, NY, USA) with vertically polarized incident light (λ = 632.8 nm) supplied by a He-Ne laser operating at 35 mW and a Brookhaven BI-9000 AT digital autocorrelator. The autocorrelation functions were analyzed using the constrained regularized algorithm CONTIN. The measurements were made at a 90° angle at 25 °C. The dispersity of particle sizes was given as $\frac{\mu_{2}}{{\bar{\Gamma }}^{2}}$, where $\bar{\Gamma }$ is the average value of the relaxation rates Γ, and *μ_2_* is its second moment. These values were obtained from cumulant analysis.

Before DLS analysis, the star polymer solutions (c = 1 mg/mL) were passed through membrane filters with nominal pore sizes of 0.2 µm (ANATOP 25 PLUS, Whatman, Maidstone, UK).

The cloud point transition temperatures of the prepared stars were determined using a Specord200 Plus (Analytik Jena, Jena, Germany) spectrophotometer equipped with a thermostated cuvette (heating rate 2 °C/min). The transmittance in DMEM culture medium and PBS (for all samples c = 1 mg/mL) was monitored at λ = 700 nm as a function of temperature. The cloud points were determined as the temperature at which the transmittance of the polymer solution reached 50% of its initial value.

In order to investigate the responsivity of the P(DMAEMA-*co*-OEGMA-OH) stars as a function of pH, the star polymers were directly dissolved in deionized water, and the pH of each solution was adjusted by adding appropriate amounts of 1 M KOH or 1 M HCl. The pH adjustments were performed using a pH meter (Elmetron, Zabrze, Poland), and the tested pH values ranged from 2 to 12.

Zeta potential measurements were performed in triplicate on a Zetasizer Nano ZS 90 (Malvern Panalytical, Malvern, UK) in disposable folded capillary cells. The adjusted solutions were equilibrated for one day at each pH value before measurement. The zeta potential (*ζ*) used in this work was calculated from the electrophoretic mobility, *u*, employing the Helmholtz–Smoluchowski Equation (1):

*u* = *εζ*/*η* (1)

where *ε* is the dielectric constant of the solvent, and *η* is the viscosity of the solvent.

## 3. Results and Discussion

### 3.1. Synthesis of Star Polymers with Poly[N,N-Dimethylaminoethyl Methacrylate-co-Hydroxyl-Bearing Oligo(Ethylene Glycol) Methacrylate] Arms (P(DMAEMA-co-OEGMA-OH))

**Scheme 1:**
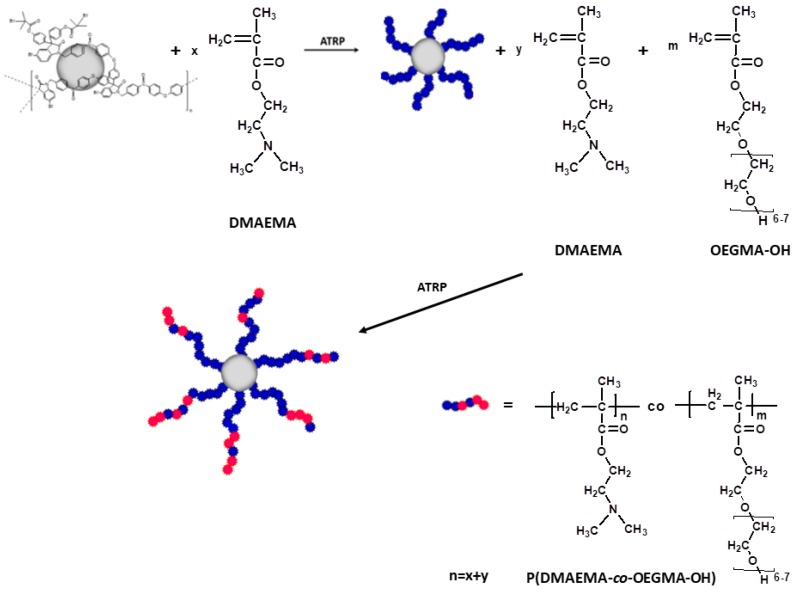
Synthesis of P(DMAEMA-*co*-OEGMA-OH) star copolymers.

Star polymers were obtained via the “core first” method using atom transfer radical polymerization (ATRP) (Scheme 1). The core of each star was a hyperbranched poly(arylene oxindole) (PArOx). The synthesis and characterization of PArOx was described previously [24]. The core provided 28 bromoester groups capable of initiating ATRP of methacrylate monomers. The absolute molar mass of PArOx, as measured by GPC-MALLS, was *M*_n_ = 21,000 g/mol and *M*_w_/*M*_n_ = 2.2 [20], while the number of initiating groups was determined from the Frey equation [25], and that value related the number of dendritic and terminal units with the degree of polymerization.

In this work, to obtain stars with copolymer arms of a desired structure, we used a different approach than we used in our earlier work on stars with random copolymer DMAEMA and di(ethylene glycol) methyl ether methacrylate arms [11]. We assumed that DMAEMA and OEGMA-OH will form arm chains with a random distribution of monomer units, as was studied by Lang et al. [26] who reported that the values of the reactivity ratios of DMAEMA and OEGMA (*M*_n_ = 475 g/mol), a monomer with a similar structure to OEGMA-OH, estimated in ATRP were close to unity. Taking this fact into consideration, after the addition of the first monomer (DMAEMA), when its conversion was respectively high, a second monomer (OEGMA-OH) was added. This “one-pot” approach ensured that the OEGMA-OH units were built in mainly at the ends of the star arms. Stars designed in this way should easily undergo post-polymerization modifications due to expected facile access to OH groups in the multiarmed and sterically crowded star structure.

The conditions for the polymerization processes are shown in Table 1.

After the optimization of ATRP conditions, the solvent, temperature, time and the initiator-to-catalyst complex ratio were the same for all polymerizations (for details, see the Experimental Section). The only variable was the initial ratio of the monomers per mole of initiating sites (Table 1). In this way, star polymers with various lengths of the arms and different contents of OEGMA-OH in the arm were obtained (Table 1).

**Figure 1:**
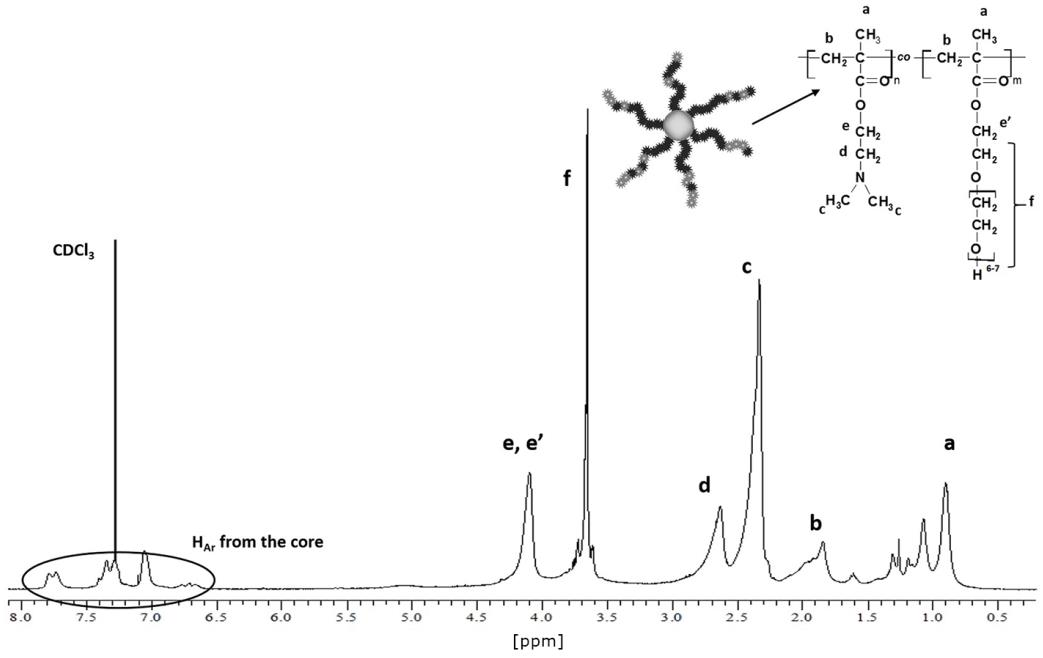
^1^H NMR spectrum (CDCl3, 600 MHz) of P(DMAEMA-*co*-OEGMA-OH) star (sample P4, Table 1).

The content of OEGMA-OH in the star arms was calculated based on the ^1^H NMR spectra taken in chloroform; the ratio of the signal of the methyl protons of the amino groups (c) in the DMAEMA to the signal of the methylene protons in the pendant chains of OEGMA-OH (f). Four stars with different molar contents of OEGMA-OH in the range from 2.6 to 10 mol% were obtained (Table 1). A representative proton NMR spectrum of a star polymer is shown in Figure 1.

The theoretical molar masses of all synthesized star polymers were calculated from the monomer conversion and compared with those obtained from GPC with multiangle laser light scattering (GPC-MALLS) detection. For GPC measurements, the refractive index increments (*dn*/*dc*) of the stars were independently measured in DMF to obtain absolute values of the molar masses (Table 2).

The theoretical molar masses of the star were calculated using Equation (2):

(2) $$M_{theor.}=M_{PArOx}+({\left[C_{DMAEMA}\right]}_{0}/{\left[C_{PArOx}\right]}_{0})\times \Delta C_{DMAEMA}\times M_{DMAEMA}\;+({\left[C_{OEGMA-OH}\right]}_{0}/{\left[C_{PArOx}\right]}_{0})\times \Delta C_{OEGMA-OH}\times M_{OEGMA-OH})\;$$

where:

*M_theor._*—the theoretical molar mass of the star, *M_PArOx_*—the molar mass of the macroinitiator, [*C_DMAEMA_*]_0_—the initial molar concentration of DMAEMA, [*C_PArOx_*]_0_—the initial molar concentration of the initiator, *ΔC_DMAEMA_*—the conversion of DMAEMA, *M_DMAEMA_*—the molar mass of DMAEMA, [*C_OEGMA_*_-*OH*_]_0_—the initial molar concentration of OEGMA-OH, Δ*C_OEGMA_*_-*OH*_—the conversion of OEGMA-OH, and *M_OEGMA_*_-*OH*_—the molar mass of OEGMA-OH.

The good correlation between the theoretical and measured molar masses (Table 2) of the obtained star polymers confirmed that the polymerization was controlled. The absolute values of the molar masses of the stars were in the range of 100,000–257,000 g/mol (Table 2).

**Figure 2:**
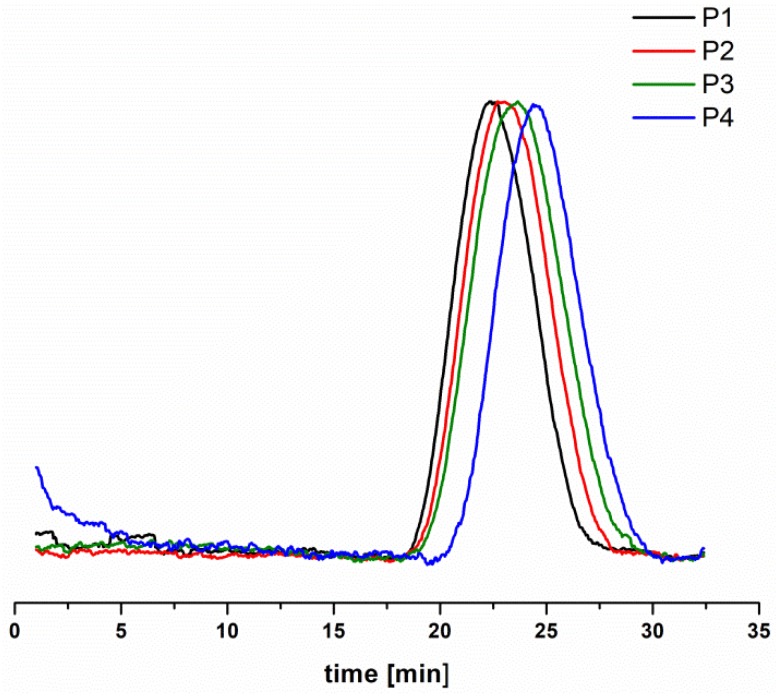
Chromatograms (RI traces) of P(DMAEMA-*co*-OEGMA-OH) star copolymers (DMF, 1 mL/min).

Very often, the synthesis of star polymers via controlled radical polymerization is accompanied by the recombination of active radical species at the ends of the star arms. This reaction often occurs when the conversion of monomers incorporated into the star arms is high; this process is called “star-star coupling” [27,28]. If this occurs, the GPC chromatograms will show a shoulder in the high molar mass region. The chromatograms of all obtained star polymers are shown in Figure 2. The peaks are monomodal and uniform. This proves that despite the relatively high DMAEMA conversions, “star-star coupling” did not occur.

### 3.2. Solution Behavior of the (P(DMAEMA-co-OEGMA-OH)) Star Copolymers

Because the size of the stars is the most important factor for biomedical applications, the relationships between the structure and solution behavior of star polymers should be examined in detail. It should be noted that in the solution, the structures of star-like macromolecules resemble the micelles formed in the self-organization of linear, amphiphilic block copolymers, and star molecules are thus often called unimolecular micelles [29]. The nomenclature of the isolated star molecules in solution and their aggregates is not uniform; in this work, the most common nomenclature found in the literature was applied.

The sizes of the obtained stars in various solvents were measured using dynamic light scattering. Five solvents were used in these experiments, two organic solvents (acetone and ethanol), pure water and two media that are often used in biomedical experiments (PBS and the most commonly used cell culture medium, DMEM). Due to the presence of various types of inorganic salts and biocompounds in culture media, such studies are necessary to properly assess the structures formed in solution.

Hydrodynamic radius (R_h_^90°^) measurements were made in triplicate at 25 °C, and the average values are summarized in Table 3. The concentration of polymers in each solvent was equal to 1 mg/mL.

Additionally, Table 3 compares the contour length of the star arms calculated for the fully extended arms by multiplying the DP of the arm and the length of one repeating unit containing two carbon atoms in the backbone (0.252 nm) [30].

The choice of solvent was mainly guided by their possible interactions with the polymer. Acetone was chosen because it is a good solvent both for the arms and the PArOx core of the stars. The remaining solvents are more selective; they do not penetrate the dense hydrophobic structure of the PArOx core, and instead, they interact with the shell formed by the arms of the star.

The obtained values of the hydrodynamic radii in acetone and PBS (Table 3) are the lowest, which suggests that in these solvents, stars exist as single macromolecules. In the others, the average values of R_h_^90°^ are slightly higher (Table 3), indicating the aggregation of a few macromolecules. We are aware, however, that comparisons between the measured values of R_h_^90°^ and the calculated contour length do not allow us to directly conclude whether the stars are isolated or if they self-organize into several interconnected structures.

**Figure 3:**
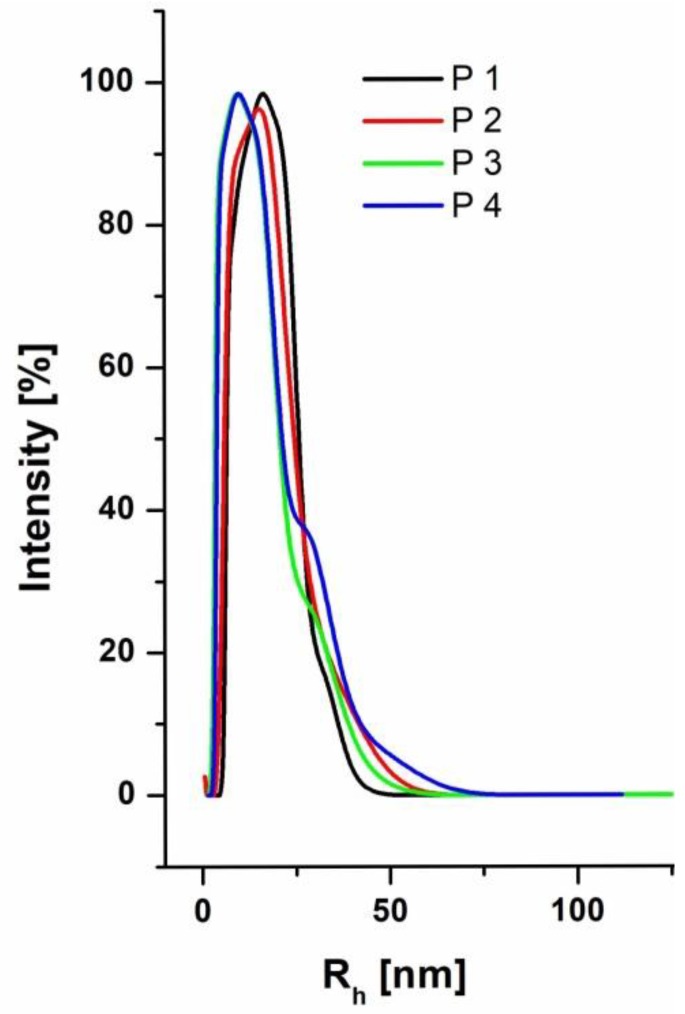
Distribution of the hydrodynamic radii of P(DMAEMA-*co*-OEGMA-OH) stars in PBS.

The representative size distributions of the star polymers (samples P1–P4, Table 1) in PBS are shown in Figure 3. The obtained results show that in the case of P3 and P4 stars, aggregates could be formed. The same trends were observed in acetone and ethanol, but in DMEM and water at the pH of dissolution, one very broad distribution was observed for all stars. However, based on the calculated contour lengths of the arms (Table 3) and the distribution of sizes (Figure 3), the formed aggregates are rather small and not the major species in the solution. Similar relationships between the size of stars in various solvents were observed for homopolymer DMAEMA stars [22] as well as for copolymer P(DMAEMA-*co*-DEGMA) stars with the PArOx core [11].

As the largest hydrodynamic diameter of the stars was in the range of fifty nanometers, we can conclude that the introduction of a new monomer, namely OEGMA-OH, only leads to limited aggregation in the solution, and that the obtained nanoparticles are still small enough for biomedical applications.

### 3.3. Temperature-Responsive Properties of the P(DMAEMA-co-OEGMA-OH) Stars

Since PDMAEMA itself is a pH- and thermoresponsive polymer [31,32], stars made of this monomer behave differently in solutions under different pH and temperature conditions [22,33,34]. The cloud point temperatures (T_CP_ values) of DMAEMA polymers depend on their molar mass and concentration as well as the charge on the amine groups in the chains [31,35]. Generally, at high pH values, weak charges on the polymer chains lead to a decrease of the observed T_CP_ values, while at low pH values, the electrostatic repulsions between the charged groups counteract the chain collapse, which increases the phase-transition temperature.

In the case of the star topology of the DMAEMA polymers, the thermoresponsive properties are much more complex because the T_CP_ may be influenced by the hydrophobicity of the core as well as the number of arms and the molar mass [31].

As the phase transition temperature is governed by the ratio of hydrophobic to hydrophilic units [36], the stars prepared in this paper are an interesting case. They possess a hydrophobic core that should decrease the T_CP_ values, but the arms of the stars consist of hydrophilic OEGMA-OH units, which should increase the transition temperature.

**Figure 4:**
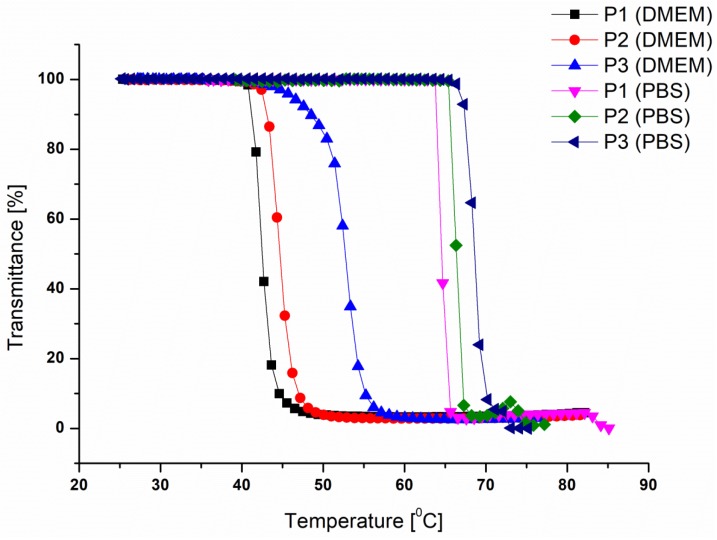
Transmittance as a function of temperature for P(DMAEMA-*co*-OEGMA-OH) stars in PBS buffer and Dulbecco’s modified Eagle’s medium (DMEM) (λ = 700 nm, c = 1 g/L).

The responses of the obtained stars to temperature changes were investigated using UV–Vis spectroscopy (Figure 4). The T_CP_ values were determined as the temperatures at which the transmittance of the polymer solution decreased to 50%. The values of the cloud point temperature for a given star differ depending on the solvent used, as shown in Table 4. It should be noted that the stars (P1–P4, Table 1) were not thermoresponsive when the pH was high enough to deprotonate the arms. The presence of the salts in aqueous solutions of PBS and DMEM enabled measurements of the phase transition temperature due to the “salting-out” effect [37]. The measurements were performed in PBS (pH = 7.4) and culture medium (DMEM), and the same sample concentration of 1 mg/mL was used in all experiments.

The phase transition temperature range of the star copolymers was between 64.5–68.5 °C in PBS and 42.4–52.8 °C in DMEM. The lower T_CP_ in DMEM (approximately 20 °C lower than in PBS) is likely caused by the larger number of different salts in DMEM than in PBS.

As mentioned above, the amount of the hydrophilic OEGMA-OH units in the star arms also influenced the T_CP_. P4 (Table 1), with the smallest molar mass and the highest content of OEGMA-OH (10 mol%), was not thermoresponsive in either PBS or DMEM. Such amount of hydrophilic monomer in the relatively short arms (Table 2) switched off the phase transition from coil to globule of the star arms, suppressing the “salting out” effect. 

In the case of polymers having a lower content of OEGMA-OH, the values of T_CP_ increase with increasing OEGMA-OH content regardless of their molar mass. Similar results were obtained by Trzebicka et al. [38] for POEGMA copolymers, and a linear increase in T_CP_ was observed with increasing content of the more hydrophilic comonomer.

The differences between the T_CP_ values of stars P1 and P2 are insignificant despite the fact that they have considerably different molar masses. The P3 sample has the highest T_CP_ because its OEGMA-OH content (4.2 mol%) (hydrophilic content) is the largest.

In our previous work, we studied the sensitivity of DMAEMA homopolymer stars with a PArOX core to temperature [22]. The thermoresponsivity of these stars was dependent on the pH of the solution, and a T_CP_ was observed only at pH 13, when the chains of the arms were unionized. Additionally, in DMEM at a neutral pH, no phase transition occurred. Here, we show that the introduction of a small fraction of OEGMA-OH into the PDMAEMA stars is sufficient to weaken the repulsive electrostatic interactions between the partially ionized arm chains and induce their collapse upon a change in temperature in DMEM.

The most suitable star polymer for further studies involving the thermoresponsive properties seemed to be star P1, as it showed with the highest molar mass and lowest OEGMA-OH content with a T_CP_ of approximately 42.4 °C, which is the closest value to physiological conditions.

### 3.4. pH Responsivity and Zeta Potential of the P(DMAEMA-co-OEGMA-OH) Stars

DMAEMA polymers are weak bases; they are fully protonated under acidic conditions and completely deprotonated under basic conditions [31,39].

We previously reported the effect of pH on the sizes of stars with PDMAEMA and P(DMAEMA-*co*-DEGMA) arms [11,22]. We observed that for PDMAEMA stars, with decreasing pH, the electrostatic repulsion from the charged amino groups stretched the arms, which resulted in an increase in the size of the nanoparticles present in the solution. At alkaline pH values, the hydrogen bonds formed between the amino groups of the PDMAEMA reduced the size of the nanostructures. The addition of DEGMA units to the star arms complicated their solution behavior as either one or two populations of nanoparticles in light scattering measurements corresponding to star and/or star aggregates were observed [11].

All P(DMAEMA-*co*-OEGMA-OH) stars are soluble in water throughout the whole pH range. For dynamic light scattering measurements, stars (samples P1–P4, Table 1) were directly dissolved in deionized water, and the pH of each solution was adjusted by adding the appropriate amount of 1 M KOH or 1 M HCl.

**Figure 5:**
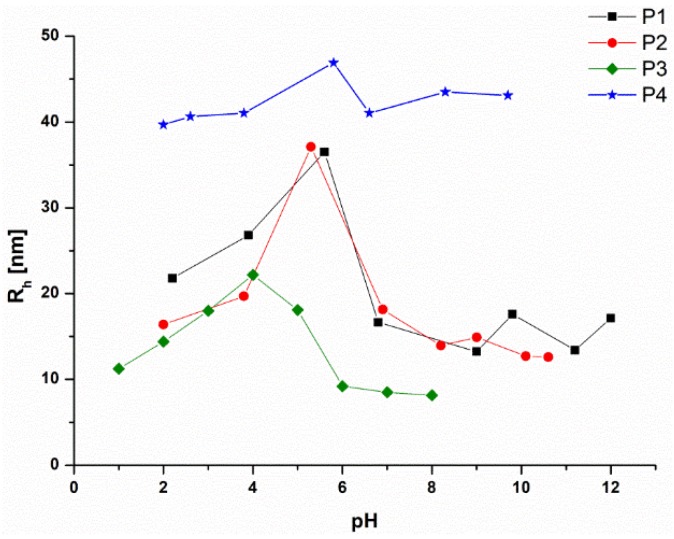
The average hydrodynamic radius as a function of pH for P(DMAEMA-*co*-OEGMA-OH) stars (the lines are guides for the eye) (c = 1 mg/mL, 90°, 25 °C).

The change in the size of the PDMAEMA star as a function of the pH of the solution is shown in Figure 5. Throughout the tested pH range, one broad distribution of particles was observed, similar to what was seen with homopolymer DMAEMA stars [22]. Nevertheless, their behavior in solution is different from that of the stars in our previous research.

Most importantly, the hydrodynamic radii of the nanoparticles do not exceed 50 nm at any of the tested pH levels, which allows their further use in biomedical applications. For all star polymers, the maximum R_h_^90°^ values were observed in the range of pH 4 to 6.9, which is probably caused by the formation of larger structures in that particular pH range.

Under low pH conditions and under high pH conditions, the sizes of P1–P3 stars remain small and most likely correspond to isolated macromolecules or aggregates of several stars. This conclusion is also supported by the lack of a definite dependency between the size of nanoparticles and the molar mass of the stars. The stars with 10 mol% OEGMA-OH reached the highest hydrodynamic radius of all tested stars (Figure 5). The maximum sizes are observed for the P4 sample, but the differences in the R_h_^90°^ values are not as great over the entire pH range as the differences seen for stars P1–P3. Based on these results, we can conclude that when the OEGMA-OH fraction is equal to or higher than 10%, it not only switches off the pH-responsive properties of the polymer but also promotes stronger aggregation compared to stars with lower contents of hydrophilic units. Based on these results, the assumption seems justified that in the case of the polymer P4 (Table 1), the switching off the temperature and pH responsivity is a complex result of the relatively high OEGMA content and the relatively small PDMAEMA fraction in the arms together with the low molar mass of the star.

For biomedical applications, the positive charge on the surface of nanoparticles facilitates their cellular uptake. This phenomenon is based upon the electrostatic interactions of the positively charged particle surfaces with the negatively charged cell membrane [12]. The zeta potential is an indicator of the surface charge of nanoparticles, and its value depends upon the pH of the solution.

**Figure 6:**
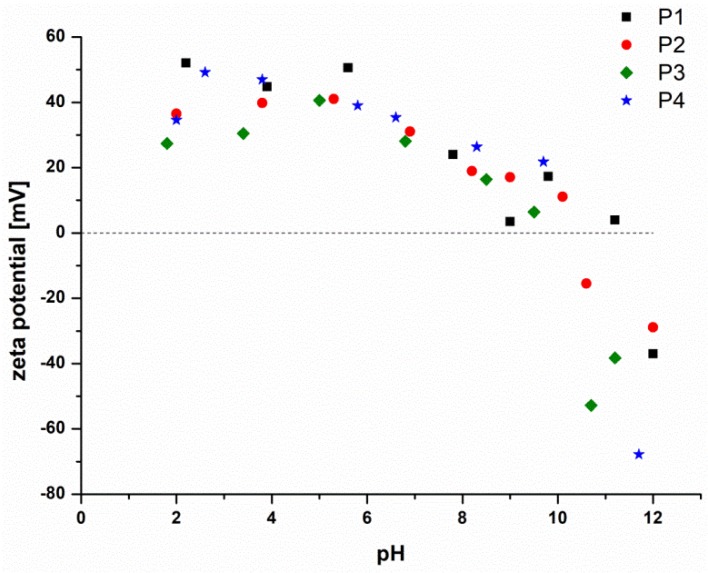
The zeta potential changes as a function of pH for P(DMAEMA-*co*-OEGMA-OH) stars. (c = 1 mg/mL, 25 °C).

For stars P1–P4, their zeta potential values decreased with increasing pH due to the loss of positively charged amino groups in the PDMAEMA (Figure 6). The change in the charge from positive to negative for all stars occurred at a pH value close to 10.

Negative zeta potential values under basic conditions were also observed for stars with copolymer arms made of DMAEMA and DEGMA [11] as well as for PDMAEMA microgels [40] and PDMAEMA chains grafted onto a poly(ethylene terephthalate) (PET) substrate [41]. This behavior is probably caused by the adsorption of negative ions by the deprotonated amine groups [40]. At pH ~7.4, which is accepted as physiological conditions, the zeta potential values are between 20 and 40 mV. Therefore, the obtained P(DMAEMA-*co*-OEGMA-OH) stars can be used in future applications, e.g., as carriers of negatively charged nucleic acids.

### 3.5. Cytotoxicity of the Obtained Star Polymers

DMAEMA polymers of different topologies have attracted significant attention as delivery systems able to transport genetic material [42] or as antimicrobial materials [43]. The cationic charge of these polymers is responsible for their interactions with anionic species, e.g., proteins present in the membranes of the cells, which results in the apoptosis and necrosis of the cells [44,45]. In the case of gene delivery, the studies have mainly focused on the cytotoxicity of complexes of PDMAEMA (both linear and stars) with nucleic acids. On the other hand, there are fewer studies on the toxicity of PDMAEMA stars themselves. The survival of the cells in the presence of stars with DMAEMA homopolymer arms has been examined, inter alia, for Chinese hamster ovary CHO-K1 [46,47] and L929 [48] cells.

**Figure 7:**
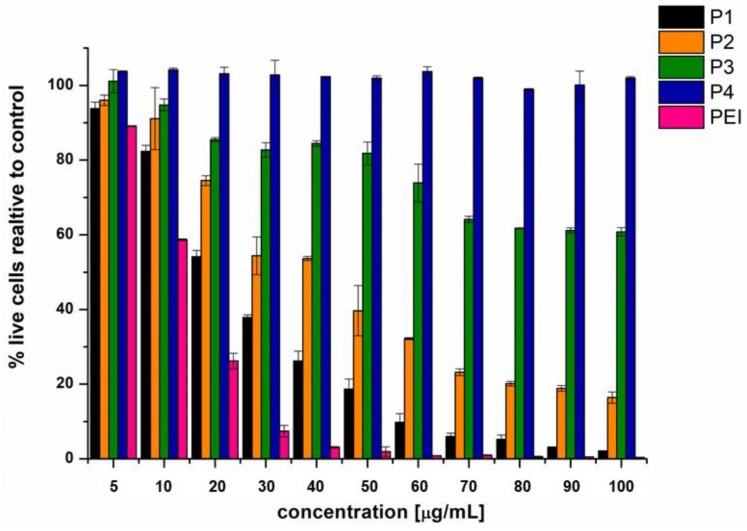
The cytotoxicity assay of the tested polymers. The assays were performed with HT-1080 cells. The results are presented as a % of cells surviving in the presence of the star polymers (P1–P4, Table 1) and branched polyethyleneimine (PEI) at increasing concentrations.

The Alamar blue reduction test was performed for HT-1080 cell line originating from a human fibrosarcoma, a malignant tumor from fibrous connective tissue. As a reference material to compare to polymers P1–P4 (Table 1), the “gold standard”—commercially available branched polyethyleneimine (PEI) was used. In all cases, it was observed that the toxicities of the stars at appropriate concentrations were much lower than that of PEI (Figure 7). The differences between the cytotoxicities of star polymers P1–P4 were mainly based on their different molar mass and chemical composition (Figure 7). For stars P1–P3 (Table 1), it may be concluded that higher concentrations of stars resulted in higher toxicities. P1 stars had the highest molar mass, as well as the highest ratio of DMAEMA:OEGMA content, and they were the most toxic (Figure 7). The introduction of the OEGMA-OH fraction resulted in a significant improvement in cell survival compared to their counterparts with PDMAEMA arms [22]. Apart from the molar mass of the star polymers, the introduction of 10% of OEGMA-OH into polymer P4 (Table 1) resulted in 100% survival of HT-1080 cells in the studied range of concentrations, and a similar effect was observed for stars with random DEGMA-*co*-OEGMA copolymer arms when the DEGMA content exceeded 40 mol% (Figure 7) [11]. The obtained results indicate that the amount of OEGMA-OH in the arms is the most important parameter for obtaining better cell viability, which should be taken into account when designing future bio-applications.

## 4. Conclusions

Star copolymers of DMAEMA and OEGMA-OH with molar masses up to 257,000 g/mol and OEGMA-OH contents up to 10 mol% were synthesized using ATRP. The introduction of the hydrophilic monomer OEGMA-OH strongly influenced the pH and thermoresponsiveness as well as the aggregation behavior of the star polymers. In organic and aqueous solutions, the stars were mainly present as isolated macromolecules, and aggregates were minor components. In general, increasing the OEGMA-OH content increased the aggregation tendency of the stars; however, as the largest hydrodynamic diameter of the stars was in the range of 100 nanometers, they still may be used in biomedical applications. The addition of 10 mol% OEGMA-OH turns off the thermoresponsive properties of the stars and limits their responsivity to changes in pH. On the other hand, the content of such a hydrophilic monomer lowers the cytotoxicity of such stars when compared to those that do not possess OEGMA-OH or have a methacrylic derivative with a methyl group instead of a hydroxyl group in their pendant ethylene glycol group. The stars obtained in this way are the least toxic at the concentrations needed for biological experiments, which is important to their applicability in biology and medicine.

## Figures and Tables

**Table 1:** Polymerization conditions for poly(*N*,*N*-dimethylaminoethyl methacrylate (DMAEMA)-*co-*hydroxyl-bearing oligo(ethylene glycol) methacrylate (OEGMA-OH)) stars.

Star	[DMAEMA]: [OEGMA]:[PArOx] ^a^	Conversion DMAEMA:OEGMA ^b^ [%]	OEGMA-OH Content ^c^ [mol%]
P1	100:10:1	44:3	2.6
P2	50:5:1	62:29	2.8
P3	30:3:1	60:56	4.2
P4	40:10:1	36:30	10.0

^a^ theoretical ratio per mol of initiating sites; ^b^ measured by HPLC; ^c^ calculated from ^1^H NMR.

**Table 2:** Molar masses of the P(DMAEMA-*co*-OEGMA-OH) star copolymers.

Star	*M*_n_^a^ [g/mol]	*dn*/*dc*^a^ [mL/g]	*M*_w_/*M*_n_^a^	M_theor_^b^ [g/mol]
P1	257 000	0.067	2.4	240 000
P2	159 000	0.074	2.5	172 000
P3	132 000	0.081	2.3	117 000
P4	100 000	0.126	2.2	114 000

^a^ from GPC-MALLS, ^b^ calculated from Equation (2).

**Table 3:** Hydrodynamic radii of the P(DMAEMA-*co*-OEGMA-OH) stars in different solvents.

Star	*M*_n_ [g/mol]	Contour Length of Arm [nm]	R_h_^90°^ [nm]				
			Acetone	EtOH	Water (pH Dissolution)	DMEM	PBS
P1	257,000	11.3	10.0 ± 2	22.4 ± 2	17.0 ± 2 (6.8)	20.0 ± 2	14.4 ± 1
P2	159,000	8.2	11.0 ± 2	20.0 ± 3	18.0 ± 1 (6.9)	19.3 ± 2	13.3 ± 2
P3	132,000	5.0	13.6 ± 1	20.5 ± 2	18.0 ± 2 (6.8)	11.7 ± 1	10.7 ± 3
P4	100,000	4.3	9.0 ± 2	26.6 ± 1	41.0 ± 2 (6.6)	27.3 ± 2	14.0 ± 1

**Table 4:** Phase transition temperature data of the P(DMAEMA-*co*-OEGMA-OH) star copolymers.

Star	*M*_n_^a^ [g/mol]	OEGMA-OH Content [mol%]	T_CP_ [°C]	
			PBS	DMEM
P1	257 000	2.6	64.5	42.4
P2	159 000	2.8	66.3	44.5
P3	132 000	4.2	68.5	52.8
